# Health literacy of adolescents using hormonal contraceptives and associated factors

**DOI:** 10.1590/0034-7167-2024-0146

**Published:** 2026-08-07

**Authors:** Marcela Ariadne Braga Gomes Tomé, Marcela Matias Sena, Mariana Cavalcante Martins, Paulo César de Almeida, Ana Larissa Gomes Machado, Fabiane do Amaral Gubert, Eveline Pinheiro Beserra, Neiva Francenely Cunha Vieira

**Affiliations:** I Universidade Federal do Ceará. Fortaleza, Ceará, Brazil; II Universidade Estadual do Ceará. Fortaleza, Ceará, Brazil; III Universidade Federal do Piauí. Teresina, Piauí, Brazil

**Keywords:** Health Literacy, Adolescent, Hormonal Contraceptive Agents, Nursing, Reproductive Health., Alfabetización en Salud, Adolescente, Agentes Anticonceptivos Hormonales, Enfermería, Salud Reproductiva.

## Abstract

**Objectives:**

to identify factors associated with the level of health literacy of adolescents using hormonal contraceptives.

**Methods:**

a descriptive, cross-sectional, and quantitative study was conducted with 147 female adolescents from five public schools. A sociodemographic questionnaire and the Health Literacy Scale-14 were administered.

**Results:**

most adolescents (80.3%) had high health literacy, but with low scores in functional literacy and high scores in communicative and critical literacy. The variables associated with health literacy were age (p=0.005), head of household (p=0.012), who they live with (p=0.009), and contraceptive use duration (p=0.007).

**Conclusions:**

adolescents demonstrated high levels of health literacy, but they initiated hormonal contraceptive use with low functional literacy, which may compromise their understanding of information about the method. Factors such as age, family composition, and contraceptive use duration influenced the level of health literacy.

## RESUMEN

**Objetivos:**

identificar los factores asociados con el nivel de alfabetización en salud de adolescentes que usan anticonceptivos hormonales.

**Métodos:**

se realizó estudio descriptivo, transversal y cuantitativo con 147 adolescentes mujeres de cinco escuelas públicas. Se administró un cuestionario sociodemográfico y la *Health Literacy Scale*-14.

**Resultados:**

la mayoría de los adolescentes (80,3%) presentó un alto nivel de alfabetización en salud, pero con puntuaciones bajas en alfabetización funcional y puntuaciones altas en los niveles comunicativo y crítico. Las variables asociadas con la alfabetización en salud fueron la edad (p=0,005), el jefe de hogar (p=0,012), con quién vive (p=0,009) y la duración del uso de anticonceptivos (p=0,007).

**Conclusiones:**

las adolescentes demostraron altos niveles de alfabetización en salud, pero iniciaron el uso de anticonceptivos hormonales con baja alfabetización funcional, lo que podría afectar su comprensión de la información sobre el método. Factores como la edad, la composición familiar y la duración del uso de anticonceptivos influyeron en el nivel de alfabetización en salud.

## INTRODUCTION

According to data from the United Nations report, in 2021, approximately 13.3 million babies were born to mothers under the age of 20. The Latin America and Caribbean region had the second highest level of adolescent fertility, with a mean rate of 53 births per 1,000 women aged 15 to 19^(1)^. The most recent data from Brazil, according to the United Nations Children’s Fund, indicate an adolescent birth rate of, on average, 43 births per 1,000 young people between the ages of 15 and 19, a figure higher than the overall mean in 2022^(2)^.

One way to prevent adolescent pregnancy is through the use of contraceptive methods, and among those available, hormonal contraceptives are widely used among adolescents. According to the Brazilian National School Health Survey (In Portuguese, *Pesquisa Nacional de Saúde do Escolar* - PeNSE), conducted in 2019 with more than 159,000 students aged 13 to 17, the contraceptive method used by most students was the birth control pill (52.6%), followed by the morning-after pill (17.3%). In the state of Ceará, taking the last sexual encounter as a reference, 60.6% of students reported using oral contraceptives, the third-highest mean in Brazil^(3)^.

However, irregular and inappropriate use of contraceptive methods is common. In a study of adolescent girls from 32 countries in Sub-Saharan Africa, participants’ reported knowledge of contraceptives was only superficial, and researchers noted that this same information could be incorrect and misleading, especially if sourced from unreliable sources. Furthermore, even with good knowledge of contraceptives, adolescents often encountered barriers to use, such as stigma, discrimination, and fear of adverse effects^(4)^.

Poor knowledge about sex education, difficulty in accessing contraceptive methods, and lack of knowledge about their correct use are associated with unfavorable sexual and reproductive health outcomes, such as low contraceptive use and adolescent pregnancy^(5)^. However, the use of contraceptives does not depend solely on correct information about them, as multiple factors influence adherence to contraception, such as the young person’s beliefs and personal motivation.

Adolescents are particularly vulnerable and, to some extent, constitute a social group with limited access to health care and health promotion services. The literature has discussed the most appropriate way to empower adolescents regarding their own health and foster the skills necessary to enable them to think critically about the health information they are exposed to, making healthy and informed choices, and striving to change the factors that constitute the determinants of health^(6,7)^.

One tool that can help in the process of understanding the correct use and greater adherence to contraceptives by sexually active adolescents is health literacy, a term defined by the World Health Organization as the ability to access, understand, assess, and use information and services, in order to promote and maintain good health and well-being for oneself and those around them, which would not be limited by the ability to read pamphlets and make appointments^(8)^.

Health literacy is understood as a variable construct that is acquired throughout life, as in a continuous learning process; therefore, the younger this construction begins, the better it is to mitigate future health risks^(9)^. Adequate health literacy skills and competencies influence health behaviors and healthy lifestyle habits that, when encouraged from childhood and adolescence, are strongly associated with better health outcomes in adulthood^(10)^. Therefore, the importance of literacy as a health promotion strategy from the earliest years is highlighted.

Although there is an increase in scientific publications related to health literacy, this topic is rarely addressed in children and adolescents in the Brazilian context, where the approach to this population’s skills and attitudes in managing their own health is still limited^(9-11)^. The study on the health literacy of adolescents using hormonal contraceptives may elucidate the factors of good or poor understanding of the appropriate use of the method and, consequently, the prevention of adolescent pregnancy and risky sexual behavior.

## OBJECTIVES

To identify factors associated with the level of health literacy of adolescents using hormonal contraceptives.

## METHODS

### Ethical aspects

The study followed the recommendations of Resolution 466/2012 of the Brazilian National Health Council, and was submitted to the *Universidade Federal do Ceará* Research Ethics Committee for review and approval. Data collection followed the guidelines for research conducted in a virtual environment, published by the Brazilian National Research Ethics Committee^(12)^. The Informed Consent Form was obtained, online or in writing, from adult adolescents and from all parents/guardians of adolescents under 18 years of age; they were asked to sign the Assent Form to allow participation in the study.

### Study design, period and location

The data analyzed in this research come from a doctoral thesis. This is a descriptive, cross-sectional study with a quantitative approach guided by STrengthening the Reporting of OBservational studies in Epidemiology recommendations. The research was conducted between January and October 2020 in five public high schools in a municipality in the state of Ceará.

### Population, sample and selection criteria

The study population consisted of all female adolescents regularly enrolled in the aforementioned institutions. The sampling process was non-probabilistic and convenience, as adolescents’ participation in the study was voluntary^(13)^. To calculate the sample size, the formula for estimating proportions^(13)^ was used, setting the confidence level at 95% and sampling error at 6%, obtaining a sample of 137 adolescents.

Inclusion criteria comprised being between 13 and 19 years old, using hormonal contraception in the last 12 months, and reporting sexual activity. Exclusion criteria included pregnant adolescents and/or those with cognitive impairments that prevented them from understanding and participating in the study.

### Study protocol

Initially, data collection took place in person at three public schools, totaling 36 questionnaires. However, with the advancement of the novel coronavirus (SARS-CoV-2) pandemic and the resulting restrictions, data collection was transferred online. The consent form, questionnaire, and scale were structured on a Google Forms^®^ platform and sent online to 111 adolescents from two other public schools, following the ethics committee’s protocol for online data collection^(12)^.

A questionnaire was applied with sociodemographic, sexual and reproductive data, with the following variables: age; marital status; family composition; number of people in the household; income; religion; menarche; occurrence of adolescent pregnancy cases in family members; sexual intercourse in the last 12 months; type of contraceptive method used; use duration; and indication for use.

Moreover, the Health Literacy Scale (HLS)-14 was used, a scale initially developed to measure the health literacy of patients with diabetes^(14)^, and later modified and expanded to the general population^(15)^. The HLS-14 was translated and validated into Brazilian Portuguese and obtained good internal consistency with a Cronbach’s alpha coefficient of 0.82^(16)^, making it a reliable instrument for use in the general population.

This instrument measures the level of health literacy based on adolescents’ reading of a medication leaflet. It has 14 questions with answers on a five-point Likert scale, ranging from “completely agree” to “completely disagree”, covering three levels: functional; communicative; and critical literacy^(16)^. To apply this instrument, the information and guidelines contained in the hormonal contraceptive package insert were considered.

The functional level corresponds to the ability to read and understand written or oral information. The communicative level refers to the ability and skills to seek information and navigate the healthcare sector, including obtaining information, negotiating, listening, and speaking. The critical level refers to the ability to make decisions, adopting behaviors that are favorable or unfavorable to health, and the power to influence others^(17)^. The cut-off score for health literacy staging in the HLS-14 is high literacy, if ≥ 46 points, and low literacy, if < 46^(16)^.

### Analysis of results and statistics

The data were tabulated and stored in a Microsoft Excel^®^ spreadsheet and later transferred to the Statistical Package for the Social Sciences version 20.0, license 10101131007, for statistical analysis.

Descriptive analyses of sociodemographic data and HLS-14 responses were performed using absolute and relative frequencies, means, and standard deviations (SD). Pearson’s chi-square test, Fisher’s exact test, and likelihood ratio were used to analyze the association between literacy level (outcome variable) and adolescents’ profile (predictor variables). The strength of the associations was analyzed using Odds Ratio with a 95% Confidence Interval. Spearman’s correlation coefficient was used to analyze the correlation between literacy scale results and numerical variables. In all statistical analyses, a significance level of 5% (*p*<0.05) was considered.

## RESULTS

The final sample had 147 participants and was predominantly composed of adolescents between 15 and 17 years old (60.5%), with a mean age of 17.4 years (SD±1.0 year), single with a steady partner (66%), whose mother was the head of household (57.8%), who lived with their father and/or mother (76.2%), with an income of up to three minimum wages (85%) and with religion (62.2%).

Concerning gynecological variables, most participants experienced menarche between the ages of 11 and 12 (51%); reported that there had been cases of adolescent pregnancy in the family (55.8%); had never been pregnant (97.3%); and had had sexual intercourse in the last 12 months (93.9%). Of the hormonal contraceptive methods, just over half used oral contraceptives (50.3%), with a mean use duration of 8.8 months (SD±8.9) (Table 1).

**Table 1:** Sociodemographic, sexual, and reproductive characteristics of adolescents using hormonal contraceptives, Fortaleza, Ceará, Brazil, 2020

Variables	n	%
**Age range (year)**	Mean ± SD: 17.4±1.0	
15 - 17	89	60.5
18 - 19	58	39.5
**Marital status**		
Married/common-law relationship	4	2.7
Single with a steady partner	97	66
Single without a partner	46	31.3
**Head of household (highest income)**		
Mother	85	57.8
Father	33	22.4
Others	29	19.7
**With whom they live**		
With father and/or mother	112	76.2
With other family members	35	23.8
**Number of people in the household**	Mean ± SD: 4.6±1.3	
2 - 4	71	48.3
5 - 8	76	51.7
**Family income (minimum wage)**	Mean ± SD: 2148.4±2266.4	
Up to 3	119	85
4 - 6	21	15
**Has religion**		
Yes	89	62.2
No	54	37.8
**Age at menarche (year)**	Mean ± SD: 11.7±1.4	
6 - 10	28	19.3
11 - 12	74	51
13 - 15	43	29.7
**Has there been any adolescent pregnancy in the family?**		
Yes	82	55.8
No	65	44.2
**If so, which family member?**		
Mother	36	24.5
Father	7	4.8
Siblings	14	9.5
Cousins	19	12.9
Aunt/grandmother	6	4.1
**Have they ever been pregnant?**		
Yes	4	2.7
No	143	97.3
**In the last 12 months, have they had sexual intercourse?**		
Yes	138	93.9
No	9	6.1
**What hormonal method do they use?**		
Oral contraceptive	74	50.3
Injectable contraceptive	35	23.8
Emergency pill	38	25.9
**Method use duration (months)**	Mean ± SD: 8.8±8.9	
1 - 8	86	63.7
9 - 36	49	36.3
**Indication for use of the method**		
Use on their own	54	37
Recommended by a healthcare professional	73	50
Recommended by family and/or friends	19	13
**Did a professional provide guidance on how to use the method?**		
Yes	69	46.9
No	78	53.1

*SD - standard deviation.*

Regarding the HLS-14 data, of the 147 adolescents assessed, 118 (80.3%) had high health literacy and 29 (19.7%) had low literacy. When separating between the dimensions (Table 2), a low level of functional literacy was found among research participants, demonstrating that more than half of adolescents had difficulty reading and understanding the hormonal contraceptive package insert.

**Table 2:** Distribution of adolescents’ responses by question on the Health Literacy Scale-14, Fortaleza, Ceará, Brazil, 2020

HLS-14 questions	Strongly agree nº (%)	Agree nº (%)	Neither agree nor disagree nº (%)	Disagree nº (%)	Strongly disagree nº (%)
**Functional literacy**					
Q1. I find characters which I cannot read.	29 (19.7)	58 (39.5)	36 (24.5)	11 (7.5)	13 (8.8)
Q2. The print is very small for me (although I use glasses).	29 (19.7)	54 (36.7)	26 (17.7)	28 (19.0)	10 (6.8)
Q3. The content is very difficult for me to understand.	26 (17.7)	33 (22.4)	39 (26.5)	32 (21.8)	17 (11.6)
Q4. It takes a long time to read (the instructions).	26 (17.7)	55 (37.4)	26 (17.7)	28 (19.0)	12 (8.2)
Q5. I need someone to help me reading them.	15 (10.2)	26 (17.7)	27 (18.4)	49 (33.3)	30 (20.4)
**Communicative literacy**					
Q6. I look for information at various sources.	52 (35.4)	69 (46.9)	18 (12.2)	4 (2.7)	4 (2.7)
Q7. I extract information that I want.	25 (17.0)	67 (45.6)	46 (31.3)	5 (3.4)	4 (2.7)
Q8. I understand the information I get.	20 (13.6)	70 (47.6)	51 (34.7)	1 (0.7)	5 (3.4)
Q9. I pass on my opinion about my CP, to the doctor, family and friends.	29 (19.7)	66 (44.9)	35 (23.8)	10 (6.8)	7 (4.8)
Q10. I apply the information I receive, through self-assessment, to my lifestyle and daily routine.	16 (10.9)	71 (48.3)	53 (36.1)	6 (4.1)	1 (0.7)
**Critical literacy**					
Q11. I take into account if the information is applicable to me.	20 (13.6)	90 (61.2)	29 (19.7)	8 (5.4)	0 (0.0)
Q12. I take into account if the information is acceptable.	33 (22.4)	94 (63.9)	19 (12.9)	1 (0.7)	0 (0.0)
Q13. I verify if the information is valid and reliable.	12 (8.2)	77 (52.4)	40 (27.2)	14 (9.5)	4 (2.7)
Q14. I put together information on the basis of my decisions about CP.	23 (15.6)	90 (61.2)	23 (15.6)	9 (6.1)	2 (1.4)

*CP - contraceptive; HLS-14 - Health Literacy Scale-14.*

However, regarding the other two levels (communicative and critical), a high level of literacy was observed among the adolescents in the study, demonstrating that the majority of participants were able to search for information in different media, analyze the information found and apply it to personal situations to adopt behaviors that were favorable or unfavorable to health.

Table 3 shows the association between adolescents’ profile and the level of health literacy. Health literacy showed a statistically significant association with the following variables: age group (*p*=0.005; OR=2.69; CI=1.34-5.37); head of household (*p*=0.012; OR=2.83; CI=1.23-6.51); with whom they live (*p*=0.009; OR=2.77; CI=1.27-6.05); and contraceptive use duration (*p*=0.007; OR=2.68; CI=1.30-5.53).

**Table 3:** Association of adolescents’ profile with health literacy level, Fortaleza, Ceará, Brazil, 2020

Variables	Low literacy nº (%)	High literacy nº (%)	OR	95%CI	*p* value
**Age range**					
18 - 19	30 (52.6)	27 (47.4)	2.69	1.34-5.37	**0.005^1^**
15 - 17	26 (29.2)	63 (70.8)	1.00		
**Marital status**					
Single with steady partner	37 (37)	63 (63)	0.83	0.40-1.70	0.619^1^
Single without steady partner	19 (41.3)	27 (58.7)	1.00		
**Head of household**					
Others	17 (58.6)	12 (41.4)	2.83	1.23-6.51	**0.012^1^**
Father/mother	39 (33.3)	78 (66.7)	1.00		
**With whom they live**					
With other family members	20 (57.1)	15 (42.9)	2.77	1.27-6.05	**0.009^1^**
With father and/or mother	36 (32.4)	75 (67.6)	1.00		
**Has religion**					
Yes	32 (36.4)	56 (63.6)	0.71	0.35-1.42	0.339^1^
No	24 (44.4)	30 (55.6)	1.00		
**Age at menarche (years)**					
6 - 10	12 (42.9)	16 (57.1)	1.04	0.39-2.72	0.934^1^
11 - 12	24 (32.9)	49 (67.1)	0.68	0.31-1.48	0.331^1^
13 - 15	18 (41.9)	25 (58.1)	1.00		
**Has there been any adolescent pregnancy in the family?**					
Yes	33 (39.8)	50 (60.2)	1.14	0.58-2.25	0.689^1^
No	23 (36.5)	40 (63.5)	1.00		
**If so, which family member?**					
Parent	20 (46.5)	23 (53.5)	1.84	0.65-5.18	0.241^1^
Sibling	5 (38.5)	8 (61.5)	1.32	0.32-5.37	0.690^1^
Other family members	8 (32)	17 (68)	1.00		
**Have they ever been pregnant?**					
Yes	2 (66.7)	1 (33.3)	3.29	0.29-37.22	0.317^2^
No	54 (37.8)	89 (62.2)	1.00		
**Have they had sexual intercourse in the last 12 months?**					
Yes	53 (38.7)	84 (61.3)	1.26	0.30-5.26	0.749^1^
No	3 (33.3)	6 (66.7)	1.00		
**What hormonal method do they use?**					
Oral	28 (38.4)	45 (61.6)	1.52	0.65-3.55	0.324^1^
Injectable	17 (48.6)	18 (51.4)	2.31	0.88-6.08	0.085^1^
Emergency pill	11 (28.9)	27 (71.1)	1.00		
**Method use duration**					
9 - 36	27 (55.1)	22 (44.9)	2.68	1.30-5.53	**0.007^1^**
1 - 8	27 (31.4)	59 (68.6)	1.00		
**Indication for use of the method**					
By family members and others	25 (34.7)	47 (65.3)	0.70	0.36-1.37	0.307^1^
By healthcare professionals	31 (42.5)	42 (57.5)	1.00		
**Have they received professional guidance on its use?**					
Yes	30 (43.5)	39 (56.5)	1.50	0.77-2.95	0.228^1^
No	26 (33.8)	51 (66.2)	1.00		

1 Pearson’s chi-square test; 2Likelihood ratio; OR - Odds Ratio; 95%CI - 95% Confidence Interval.

A higher frequency of high health literacy was observed among single women with a steady partner (84.2%; *p*=0.116; OR=2.0; CI=0.9-4.8), among young women who had adolescent pregnancies in their families (60.2%; *p*=0.689; OR=1.14; CI=0.5-2.2), as well as among participants who did not become pregnant (62.2%; *p*=0.317; OR=3.2; CI=0.2-37.2). Regarding the type of hormonal contraceptive used, the three methods showed a majority of participants with high literacy.

Based on the *p*-values of Spearman’s correlation test, when crossed with numerical variables (Table 4), it was found that the HLS-14 total score correlated in an inversely proportional manner only with the age variable (rs=-0.218; *p*=0.008).

**Table 4:** Linear correlation analysis using Spearman’s correlation coefficient between the Health Literacy Scale-14 score and numerical variables, Fortaleza, Ceará, Brazil, 2020

Variables	HLS-14 total score	
	rs	*p* value
How old are you? (n=146)	-0.218	**0.008**
How many people live in the household? (n=146)	-0.045	0.591
How old were you when you got your first period? (n=144)	0.021	0.798
Method (current) use duration in months (n=135)	-0.160	0.063

*rs - Spearman’s rho; HLS-14 - Health Literacy Scale-14.*

## DISCUSSION

The research sample consisted of sexually active adolescents who used hormonal contraception, with girls beginning their sexual relations at an early age, starting at 15. The mean age was higher than that found in the PeNSE-2019 study, which assessed students from public and private schools nationwide and found that 35.4% of students between the ages of 13 and 17 had had sexual intercourse at some point, with a mean age of first sexual intercourse of 13.8 years^(3)^.

The non-use of contraceptive methods, the fact of being single and the lack of support from the child’s father were associated with the occurrence of adolescent pregnancy in a study carried out with 518 pregnant women from São Tomé and Príncipe, a country in Central Africa, in which, of these, 20.1% were adolescents^(18)^. In the present study, 50.3% of participants reported using oral contraceptives, and 25.9% reported using the emergency pill as a contraceptive method, showing a worrying percentage of adolescents who use the morning-after pill as a regular method, which compromises its effectiveness.

Inconsistent/inadequate use of contraceptive methods increases their failure rate and the chance of an unwanted pregnancy, and increases the risk of maternal morbidity and mortality, according to research conducted in Nigeria^(19)^. In a study conducted in Kenya, it was found that adolescents and young women (15 to 24 years old) had a 1.2 times greater risk of stopping the use of a contraceptive method and showed a 20% increase in the risk of discontinuing a method during the period analyzed, when compared to the group of women over 35 years old^(20)^.

Given the above, the question is how adolescents have understood the information they receive, what sources of information they have sought, and what basis they have used to make decisions. Preventing unwanted and/or unplanned pregnancies in adolescence requires access to accurate information, adequate guidance, and critical judgment to guide decision-making.

In this study, most adolescents (80.3%) had high health literacy, which favors the adoption of preventive measures and safe health behaviors. Similarly, in studies conducted in Norway and South Korea, adolescents had high and moderate levels of health literacy^(21,22)^. In these studies, health literacy was associated with health protection measures and healthy behaviors related to the COVID-19 pandemic.

On the other hand, a study carried out in Ethiopia with 577 high school students found that the majority of adolescents had poor knowledge about condom use and sexually transmitted diseases, and 69.6% of adolescents had a problematic or inadequate score in sexual and reproductive health literacy, similar to that observed in a Brazilian study, in which 69.44% of students had low levels of health literacy related to contraceptive methods^(23,24)^.

The adolescents in this study had low levels of functional literacy, but high levels of communicative and critical literacy. This situation is characterized by low reading skills, poor comprehension of written information, and poor understanding of oral instructions given by healthcare professionals, which impairs decision-making and leads to, for instance, the incorrect use of medications^(17)^.

Reading and numeracy skills, components of functional literacy, are essential for acquiring health literacy, including basic information acquisition skills. In Indonesia, in a study of 1,066 students aged 14 to 19, only 25% of participants had a high level of functional health literacy, and adequate scores in this dimension were associated with health behaviors such as avoiding alcohol, drug, and tobacco use^(25)^.

A survey of 288 Brazilian adolescents in a municipality in the state of Pará found that the majority reported difficulty understanding written guidance, as well as problems understanding the content of educational materials on contraceptive methods. Furthermore, 58.6% also reported difficulty understanding verbal guidance provided by healthcare professionals^(24)^.

In relation to age, an inversely proportional correlation was found with health literacy (rs=-0.218; *p*=0.008), in which adolescents between 18 and 19 years old were 2.69 times more likely to have low literacy, when compared to younger ones (*p*=0.005). This finding is consistent with that of previous studies conducted in states in southeastern Brazil, in which younger age was associated with higher levels of functional health literacy, along with higher education and high income^(26,27)^.

Similarly, a study conducted in China with different age groups found that health literacy declined as age advanced, with adolescents showing better literacy levels when compared to other age groups^(28)^. With more frequent contact with the internet and social networks, young people, in general, are more likely to seek and find health information, but have greater difficulty in assessing its veracity and safety, which influences their level of health literacy^(24,29)^.

Regarding family variables, adolescents were more likely to have low literacy when the head of household (the person with the highest wage) was not their father or mother (OR=2.83; *p*=0.012) and when they did not live with either parent (OR=2.77; *p*=0.009).

In research conducted with adolescents and young women in Mato Grosso, Brazil, participants who did not belong to nuclear families were more likely to become pregnant in adolescence, when compared to young women from families with two parents^(30)^. This family composition was also associated with risky sexual behavior, early sexual initiation in a national study conducted with Brazilian adolescents^(31)^ and excessive alcohol consumption among young people in a municipality in Bahia^(32)^.

Regarding contraceptive use, the only variable that showed a significant association with health literacy was method use duration, in which adolescents who had been using contraceptives for a longer time were 2.68 times more likely to have low literacy than those who had been using them for a shorter time (*p*=0.007), a fact similar to that found in a study carried out with 52 women using injectable hormonal contraceptives, in which the majority of women using the method unsafely had been using it for more than 12 months^(33)^.

Contraceptive method duration use and discontinuation were related to several factors, such as lack of access, little knowledge about how to deal with the method and the emergence of side effects^(20,33)^, with adequate guidance and guidance during reproductive planning consultations regarding the management of the method being fundamental, ensuring patients’ understanding of its correct use.

Studies show that people with greater health literacy are more likely to adopt healthier behaviors because they have a greater ability to understand the guidance given to them, such as prescriptions, appointment scheduling, medication labels, and other self-care information^(16)^.

### Study limitations

The study limitations are related to the sample size and the smaller age range, which occurred due to data collection difficulties arising from the pandemic, making it difficult to generalize the study to schools with different realities. Furthermore, some school principals were reluctant to participate in the study due to the topic of contraceptive use, as they reported fears that parents would consider it an incentive to engage in sexual activity, a fact that also contributed to a decrease in adolescents’ participation in the study.

### Contributions to nursing, health or public policy

Understanding the factors that contribute to the correct use of contraceptives allows healthcare professionals to develop communication strategies that facilitate informed decision-making and encourage greater autonomy in the care process for young people. Studies of this nature expand the body of knowledge for strengthening nursing in evidence-based practice, with an emphasis on the use of educational strategies that promote the use and appropriate adherence to contraceptive methods among adolescents.

## CONCLUSIONS

The participating adolescents demonstrated high levels of health literacy, reflecting their ability to seek and assess information about reproductive health. However, in contrast, it was observed that they initiated hormonal contraceptive use and their sexual life with functional literacy deficits, i.e., low reading and comprehension skills regarding the method. This gap represents a significant risk factor, as it can lead to misinterpretation of guidelines, incorrect contraceptive use, and, consequently, an increased likelihood of unwanted and/or unplanned pregnancy.

Although they demonstrated good performance in communication and critical thinking, functional literacy deficits limited their autonomy and self-care. Low health literacy was associated with age, family composition (head of household, with whom they live), and contraceptive use duration, suggesting that, in addition to intrinsic factors, family environment and prior experience also affect health behaviors.

Given these findings, it is urgent to advance research on adolescent health literacy in Brazil, not only in the context of contraceptive use, but also in other topics relevant to this population, aiming to guide and support professional practices in nursing and public health, and allowing the creation of educational strategies adapted to them.

## Data Availability

The research data are available in a repository: https://osf.io/jmhbg/?view_only=ab0331e8f13e4ff0b0cb232fb68c6c21.
