# Supplementary material for: Health literacy of adolescents using hormonal contraceptives and associated factors
Source: Rev Bras Enferm. 2026 Aug 7;79:e20240146. doi: 10.1590/0034-7167-2024-0146 (PMC13450939; doi:10.1590/0034-7167-2024-0146)
Supplement: Translation

# Translation: Letramento em saúde de adolescentes em uso de anticoncepcionais hormonais e fatores associados

**DOI:** 10.1590/0034-7167-2024-0146pt

**Authors:** Marcela Ariadne Braga Gomes Tomé, Marcela Matias Sena, Mariana Cavalcante Martins, Paulo César de Almeida, Ana Larissa Gomes Machado, Fabiane do Amaral Gubert, Eveline Pinheiro Beserra, Neiva Francenely Cunha Vieira

**Affiliations:** I Universidade Federal do Ceará. Fortaleza, Ceará, Brasil; II Universidade Estadual do Ceará. Fortaleza, Ceará, Brasil; III Universidade Federal do Piauí. Teresina, Piauí, Brasil

**Keywords:** Letramento em Saúde, Adolescente, Contraceptivos Hormonais, Enfermagem, Saúde Reprodutiva.

## Abstract

**Objetivos:**

identificar os fatores associados ao nível de letramento em saúde de adolescentes em uso de anticoncepcionais hormonais.

**Métodos:**

realizou-se estudo descritivo, transversal e quantitativo com 147 adolescentes do sexo feminino de cinco escolas públicas. Foram aplicados um questionário sociodemográfico e a *Health Literacy Scale*-14.

**Resultados:**

a maioria das adolescentes (80,3%) apresentou alto letramento em saúde, entretanto com baixa pontuação no letramento funcional e alta pontuação nos níveis comunicativo e crítico. As variáveis associadas ao letramento em saúde foram idade (*p*=0,005), chefe da família (*p*=0,012), com quem mora (*p*=0,009) e tempo de uso do método anticoncepcional (*p*=0,007).

**Conclusões:**

as adolescentes demonstraram alto nível de letramento em saúde, mas iniciaram o uso de anticoncepcionais hormonais com baixo letramento funcional, o que pode comprometer a compreensão das informações sobre o método. Fatores como idade, composição familiar e tempo de uso do anticoncepcional influenciaram o nível de letramento em saúde.

## INTRODUÇÃO

Segundo dados do relatório da Organização das Nações Unidas, no ano de 2021, cerca de 13,3 milhões de bebês nasceram de mães com idade menor do que 20 anos. A região da América Latina e Caribe obteve o segundo maior nível de fertilidade na adolescência, com taxa média de 53 nascimentos por 1.000 mulheres com idade entre 15 e 19 anos^(1)^. Os dados mais recentes do Brasil, de acordo com o Fundo das Nações Unidas para a Infância, apontam um percentual de natalidade na adolescência de, em média, 43 nascimentos por 1.000 jovens entre 15 e 19 anos, valor maior que a média global no ano de 2022^(2)^.

Uma das formas de prevenção da gravidez na adolescência é o uso de métodos contraceptivos e, entre os disponíveis, os anticoncepcionais hormonais são bastante difundidos entre as adolescentes. De acordo com a Pesquisa Nacional de Saúde do Escolar (PeNSE), realizada em 2019, com mais de 159 mil escolares entre 13 e 17 anos, o método contraceptivo utilizado pela maioria dos estudantes foi a pílula anticoncepcional (52,6%), seguida pela pílula do dia seguinte (17,3%). No estado do Ceará, tomando como referência a última relação sexual, 60,6% dos estudantes destacaram o uso do anticonceptivo oral, apresentando a terceira maior média do Brasil^(3)^.

No entanto, é comum observar o uso irregular e inadequado dos métodos. Em estudo realizado com adolescentes mulheres de 32 países da África Subsaariana, o conhecimento reportado pelas participantes sobre contraceptivos era apenas superficial, e os pesquisadores observaram que essas mesmas informações poderiam estar incorretas e equivocadas, principalmente se provenientes de meios não confiáveis. Além disso, mesmo com bom conhecimento sobre contraceptivos, as adolescentes frequentemente encontravam barreiras para o uso, como estigma, discriminação e medo dos efeitos adversos^(4)^.

O pouco conhecimento acerca de educação sexual, a dificuldade de acesso a métodos contraceptivos e o desconhecimento sobre o uso correto deles estão associados a resultados desfavoráveis de saúde sexual e reprodutiva, como o baixo uso de contraceptivos e gravidez na adolescência^(5)^. Porém, a utilização do contraceptivo não depende apenas de informações corretas sobre ele, pois múltiplos fatores exercem influência na adesão à contracepção, como as crenças e a motivação pessoal do jovem.

Os adolescentes são especialmente vulneráveis e, em certa medida, constituem um grupo social que pouco tem acesso às unidades de saúde para as ações de cuidado e promoção de saúde. A literatura tem discutido sobre a maneira mais adequada de promover o empoderamento do adolescente em relação à própria saúde e estimular as habilidades necessárias para torná-lo capaz de pensar criticamente acerca das informações de saúde a que estão expostos, fazendo escolhas saudáveis e bem-informadas, e empenhando-se em mudar os fatores que constituem os determinantes de saúde^(6,7)^.

Uma ferramenta que pode auxiliar no processo de compreensão do uso correto e maior adesão do contraceptivo por adolescentes sexualmente ativas é o letramento em saúde (*health literacy*), termo definido pela Organização Mundial da Saúde como a capacidade de ter acesso, compreender, avaliar e utilizar informações e serviços, de forma a promover e manter a boa saúde e o bem-estar para si e para aqueles ao seu redor, o que não seria limitado pela capacidade de ler panfletos e marcar consultas^(8)^.

Entende-se o letramento em saúde como construto variável que é adquirido ao longo da vida, como em um processo de aprendizagem contínuo; logo, quanto mais jovem se inicia esta construção, melhor é para amenizar os riscos futuros para a saúde^(9)^. Habilidades e competências adequadas do letramento em saúde influenciam comportamentos de saúde e hábitos de vida saudáveis que, quando estimulados desde a infância e adolescência, estão fortemente associados a melhores resultados de saúde na vida adulta^(10)^. Destaca-se, assim, a importância do letramento como estratégia de promoção da saúde desde os primeiros anos.

Embora haja um crescimento nas publicações científicas relacionadas ao letramento em saúde, essa temática é pouco abordada em crianças e adolescentes no contexto brasileiro, em que a abordagem sobre as competências e atitudes dessa população no manejo da própria saúde ainda é limitada^(9-11)^. O estudo sobre o letramento em saúde de adolescentes em uso de anticoncepcionais hormonais poderá elucidar os fatores de boa ou má compreensão do uso adequado do método e, consequentemente, a prevenção da gravidez na adolescência e de comportamentos sexuais de risco.

## OBJETIVOS

Identificar os fatores associados ao nível de letramento em saúde de adolescentes em uso de anticoncepcionais hormonais.

## MÉTODOS

### Aspectos éticos

O estudo seguiu as recomendações da Resolução n^o^ 466/2012 do Conselho Nacional de Saúde, e foi submetido à apreciação do Comitê de Ética em Pesquisa da Universidade Federal do Ceará, recebendo aprovação. A coleta de dados seguiu as diretrizes para pesquisas realizadas em ambiente virtual, publicadas pela Comissão Nacional de Ética em Pesquisa^(12)^. O Termo de Consentimento Livre e Esclarecido foi obtido, por *meio* online ou escrito, junto às adolescentes maiores de idade e de todos os pais/responsáveis das adolescentes menores de 18 anos; a essas, solicitou-se a assinatura do Termo de Assentimento para permitir a participação no estudo.

### Desenho, período e local do estudo

Os dados analisados nesta pesquisa são oriundos de tese de doutorado. Trata-se de estudo descritivo, com delineamento transversal e abordagem quantitativa norteada segundo as recomendações do *STrengthening the Reporting of OBservational studies in Epidemiology.* A pesquisa foi realizada entre janeiro e outubro de 2020 em cinco escolas do ensino médio da rede pública de um município do estado do Ceará.

### População, amostra e critérios de seleção

A população do estudo foi composta por todas as adolescentes do sexo feminino regularmente matriculadas nas referidas instituições. O processo de amostragem foi não probabilística e por conveniência, pois a adesão das adolescentes ao estudo foi espontânea^(13)^. Para o cálculo do tamanho amostral, foi utilizada a fórmula para estimação de proporção^(13)^, fixando-se o nível de confiança em 95% e erro amostral em 6%, obtendo uma amostra de 137 adolescentes.

Os critérios de inclusão foram possuir entre 13 e 19 anos, fazer uso de anticoncepcional hormonal nos últimos 12 meses e declarar-se sexualmente ativa. Os critérios de exclusão foram ser adolescentes grávidas e/ou com alguma limitação cognitiva que impossibilitasse a compreensão e participação no estudo.

### Protocolo do estudo

Inicialmente, a coleta de dados ocorreu de forma presencial em três escolas públicas, totalizando 36 questionários aplicados. Porém, com o avanço da pandemia do novo coronavírus (SARS-CoV-2) e das restrições advindas desse processo, a coleta foi aplicada de maneira *online*. O termo de consentimento, o questionário e a escala foram estruturados para um formulário da plataforma *Google Forms* ^®^, e enviados de forma *online* para 111 adolescentes de outras duas escolas públicas, seguindo o protocolo do comitê de ética para a coleta de dados por meio virtual^(12)^.

Aplicou-se questionário com dados sociodemográficos, sexuais e reprodutivos com as seguintes variáveis: idade; estado conjugal; composição familiar; número de pessoas na residência; renda; religião; menarca; ocorrência de casos de gravidez na adolescência de familiares; relação sexual nos últimos 12 meses; tipo de método contraceptivo utilizado; tempo de uso; e indicação de uso.

Além disso, foi utilizada a *Health Literacy Scale* (HLS)-14, escala desenvolvida inicialmente para mensurar o letramento em saúde de pacientes com diabetes^(14)^, sendo posteriormente modificada e ampliada para a população geral^(15)^. A HLS-14 foi traduzida e validada para o português do Brasil, e obteve uma boa consistência interna com o coeficiente alfa de Cronbach de 0,82^(16)^, sendo um instrumento confiável para uso na população geral.

Esse instrumento mensura o nível de letramento em saúde a partir da leitura que a adolescente faz da bula de um medicamento. Possui 14 perguntas com respostas dentro da escala Likert de cinco pontos, variando de “concordo totalmente” a “discordo totalmente”, contemplando três níveis: letramento funcional; comunicativo; e crítico^(16)^. Para aplicação desse instrumento, foram consideradas as informações e orientações contidas na bula dos anticoncepcionais hormonais.

O nível funcional corresponde à habilidade de leitura e compreensão de informações escritas ou passadas de maneira oral. O nível comunicativo se refere à habilidade e competência de buscar informações, e transitar no setor da saúde, a fim de obter informações, negociar, escutar e falar. Já o nível crítico diz respeito à capacidade de tomada de decisão, à adoção de comportamentos favoráveis ou não à saúde e ao poder de influenciar outras pessoas^(17)^. A nota de corte para o estadiamento da literacia em saúde na HLS-14 é alto letramento, se ≥ 46 pontos, e baixo letramento, se < 46^(16)^.

### Análise dos resultados e estatística

Os dados foram tabulados e armazenados em planilha do *Microsoft Excel* ^®^, e, posteriormente, transferidos para o programa *Statistical Package for the Social Sciences*, versão 20.0, licença 10101131007, para análise estatística.

Foram realizadas análises descritivas dos dados sociodemográficos e das respostas da HLS-14 por meio de frequências absolutas e relativas, médias, e desvios padrão (DP). Para análise da associação do nível de letramento (variável desfecho) com o perfil das adolescentes (variáveis preditoras), empregaram-se o teste qui-quadrado de Pearson, teste exato de Fisher e razão de verossimilhança. A força das associações foi analisada pelas razões de chances com Intervalo de Confiança de 95%. Para analisar a correlação entre o resultado da escala de letramento e as variáveis numéricas, foi utilizado o coeficiente de correlação de Spearman. Em todas as análises estatísticas, foi considerado o nível de significância de 5% (p < 0,05).

## RESULTADOS

A amostra final contou com 147 participantes, e foi composta predominantemente por adolescentes entre 15 e 17 anos (60,5%), com média de idade de 17,4 anos (DP±1,0 ano), solteiras com parceiro fixo (66%), cuja mãe era a chefe da família (57,8%), que morava com o pai e/ou a mãe (76,2%), com renda de até três salários mínimos (85%), e com religião (62,2%).

Quanto às variáveis ginecológicas, a maioria das participantes passou pela menarca entre 11 e 12 anos (51%); relatou que na família já houve casos de gravidez na adolescência (55,8%); nunca engravidou (97,3%); e teve relações sexuais nos últimos 12 meses (93,9%). Dos métodos anticoncepcionais hormonais, pouco mais da metade fazia uso do anticoncepcional oral (50,3%), com média de tempo de uso de 8,8 meses (DP±8,9) (Tabela 1).

**Tabela 1:** Caracterização sociodemográfica, sexual e reprodutiva das adolescentes que usam anticoncepcional hormonal, Fortaleza, Ceará, Brasil, 2020

Variáveis	n	%
**Faixa etária (ano)**	Média ± DP: 17,4±1,0	
15 - 17	89	60,5
18 - 19	58	39,5
**Estado civil**		
Casado/união estável	4	2,7
Solteiro com parceiro fixo	97	66
Solteiro sem parceiro	46	31,3
**Chefe da família (maior renda)**		
Mãe	85	57,8
Pai	33	22,4
Outros	29	19,7
**Com quem reside**		
Com pai e/ou mãe	112	76,2
Com outros familiares	35	23,8
**Número de pessoas na residência**	Média ± DP: 4,6±1,3	
2 - 4	71	48,3
5 - 8	76	51,7
**Renda familiar (salário mínimo)**	Média ± DP: 2148,4±2266,4	
Até 3	119	85
4 - 6	21	15
**Possui religião**		
Sim	89	62,2
Não	54	37,8
**Idade da menarca (ano)**	Média ± DP: 11,7±1,4	
6 - 10	28	19,3
11 - 12	74	51
13 - 15	43	29,7
**Na família já ocorreu gravidez na adolescência?**		
Sim	82	55,8
Não	65	44,2
**Caso sim, qual parente?**		
Mãe	36	24,5
Pai	7	4,8
Irmãos	14	9,5
Primos	19	12,9
Tia/avó	6	4,1
**Já engravidou?**		
Sim	4	2,7
Não	143	97,3
**Nos últimos 12 meses, teve relação sexual?**		
Sim	138	93,9
Não	9	6,1
**Qual método hormonal você usa?**		
Anticoncepcional oral	74	50,3
Anticoncepcional injetável	35	23,8
Pílula de emergência	38	25,9
**Tempo de uso do método (meses)**	Média ± DP: 8,8±8,9	
1 - 8	86	63,7
9 - 36	49	36,3
**Indicação de uso do método**		
Usa por conta própria	54	37
Indicado por profissional de saúde	73	50
Indicado por familiares e/ou amigos	19	13
**O profissional orientou quanto à forma de uso do método?**		
Sim	69	46,9
Não	78	53,1

*DP - desvio padrão.*

Com relação aos dados da HLS-14, das 147 adolescentes avaliadas, 118 (80,3%) possuíam alto letramento em saúde, e 29 (19,7%), baixo letramento. Ao separar entre as dimensões (Tabela 2), constatou-se baixo nível de letramento funcional entre as participantes da pesquisa, demonstrando que mais da metade das adolescentes tinha dificuldade de ler e compreender a bula do anticoncepcional hormonal.

**Tabela 2:** Distribuição das respostas das adolescentes por questão da *Health Literacy Scale*-14, Fortaleza, Ceará, Brasil, 2020

Questões da HLS-14	Concordo muito nº (%)	Concordo nº (%)	Nem concordo, nem discordo nº (%)	Discordo nº (%)	Discordo muito nº (%)
**Letramento funcional**					
Q1. Eu encontro palavras que não consigo ler.	29 (19,7)	58 (39,5)	36 (24,5)	11 (7,5)	13 (8,8)
Q2. A letra é muito pequena para mim.	29 (19,7)	54 (36,7)	26 (17,7)	28 (19,0)	10 (6,8)
Q3. O conteúdo é muito difícil de entender.	26 (17,7)	33 (22,4)	39 (26,5)	32 (21,8)	17 (11,6)
Q4. Demoro muito para ler (as instruções).	26 (17,7)	55 (37,4)	26 (17,7)	28 (19,0)	12 (8,2)
Q5. Eu preciso que alguém me ajude a ler.	15 (10,2)	26 (17,7)	27 (18,4)	49 (33,3)	30 (20,4)
**Letramento comunicativo**					
Q6. Eu procuro informação em vários lugares.	52 (35,4)	69 (46,9)	18 (12,2)	4 (2,7)	4 (2,7)
Q7. Eu encontro a informação que preciso.	25 (17,0)	67 (45,6)	46 (31,3)	5 (3,4)	4 (2,7)
Q8. Eu entendo a informação encontrada.	20 (13,6)	70 (47,6)	51 (34,7)	1 (0,7)	5 (3,4)
Q9. Eu falo minha dúvida sobre o uso do AC ao profissional da saúde, familiares ou amigos.	29 (19,7)	66 (44,9)	35 (23,8)	10 (6,8)	7 (4,8)
Q10. Eu coloco em prática as informações encontradas no meu dia a dia.	16 (10,9)	71 (48,3)	53 (36,1)	6 (4,1)	1 (0,7)
**Letramento crítico**					
Q11. Eu sei quando as informações são boas no meu caso.	20 (13,6)	90 (61,2)	29 (19,7)	8 (5,4)	0 (0,0)
Q12. Eu levo em conta se as informações são verdadeiras.	33 (22,4)	94 (63,9)	19 (12,9)	1 (0,7)	0 (0,0)
Q13. Eu tenho conhecimento para julgar se as informações são confiáveis.	12 (8,2)	77 (52,4)	40 (27,2)	14 (9,5)	4 (2,7)
Q14. Eu pego informações que me ajudam a tomar decisões do uso de AC.	23 (15,6)	90 (61,2)	23 (15,6)	9 (6,1)	2 (1,4)

*AC - anticoncepcional; HLS-14 - Health Literacy Scale-14.*

Entretanto, a respeito dos outros dois níveis (comunicativo e crítico), observou-se alto nível de literacia entre as adolescentes do estudo, demonstrando que a maioria das participantes conseguia buscar informações nos diferentes meios de comunicação, analisar as informações encontradas e aplicá-las a situações pessoais para adoção de comportamentos favoráveis ou não à saúde.

A associação do perfil das adolescentes com o nível de letramento em saúde está descrita na Tabela 3. O letramento em saúde obteve associação estatisticamente significante com as seguintes variáveis: faixa etária (*p*=0,005; RC=2,69; IC=1,34-5,37); chefe da família (*p*=0,012; RC=2,83; IC=1,23-6,51); com quem mora (*p*=0,009; RC=2,77; IC=1,27-6,05); e tempo de uso do método contraceptivo (*p*=0,007; RC=2,68; IC=1,30-5,53).

**Tabela 3:** Associação do perfil das adolescentes com o nível de letramento em saúde, Fortaleza, Ceará, Brasil, 2020

Variáveis	Letramento baixo nº (%)	Letramento alto nº (%)	RC	IC95%	Valor de *p*
**Faixa etária**					
18 - 19	30 (52,6)	27 (47,4)	2,69	1,34-5,37	**0,005^1^**
15 - 17	26 (29,2)	63 (70,8)	1,00		
**Estado conjugal**					
Solteira com parceiro fixo	37 (37)	63 (63)	0,83	0,40-1,70	0,619^1^
Solteira sem parceiro fixo	19 (41,3)	27 (58,7)	1,00		
**Chefe da família**					
Outros	17 (58,6)	12 (41,4)	2,83	1,23-6,51	**0,012^1^**
Pai/mãe	39 (33,3)	78 (66,7)	1,00		
**Com quem reside**					
Com outros familiares	20 (57,1)	15 (42,9)	2,77	1,27-6,05	**0,009^1^**
Com pai e/ou mãe	36 (32,4)	75 (67,6)	1,00		
**Possui religião**					
Sim	32 (36,4)	56 (63,6)	0,71	0,35-1,42	0,339^1^
Não	24 (44,4)	30 (55,6)	1,00		
**Idade da menarca (anos)**					
6 - 10	12 (42,9)	16 (57,1)	1,04	0,39-2,72	0,934^1^
11 - 12	24 (32,9)	49 (67,1)	0,68	0,31-1,48	0,331^1^
13 - 15	18 (41,9)	25 (58,1)	1,00		
**Na família, já ocorreu gravidez na adolescência?**					
Sim	33 (39,8)	50 (60,2)	1,14	0,58-2,25	0,689^1^
Não	23 (36,5)	40 (63,5)	1,00		
**Caso sim, qual parente?**					
Pai/mãe	20 (46,5)	23 (53,5)	1,84	0,65-5,18	0,241^1^
Irmão/irmã	5 (38,5)	8 (61,5)	1,32	0,32-5,37	0,690^1^
Outros familiares	8 (32)	17 (68)	1,00		
**Já engravidou?**					
Sim	2 (66,7)	1 (33,3)	3,29	0,29-37,22	0,317^2^
Não	54 (37,8)	89 (62,2)	1,00		
**Nos últimos 12 meses, teve relação sexual?**					
Sim	53 (38,7)	84 (61,3)	1,26	0,30-5,26	0,749^1^
Não	3 (33,3)	6 (66,7)	1,00		
**Qual método hormonal você usa?**					
Oral	28 (38,4)	45 (61,6)	1,52	0,65-3,55	0,324^1^
Injetável	17 (48,6)	18 (51,4)	2,31	0,88-6,08	0,085^1^
Pílula de emergência	11 (28,9)	27 (71,1)	1,00		
**Tempo de uso do método**					
9 - 36	27 (55,1)	22 (44,9)	2,68	1,30-5,53	**0,007^1^**
1 - 8	27 (31,4)	59 (68,6)	1,00		
**Indicação de uso do método**					
Por familiares e outros	25 (34,7)	47 (65,3)	0,70	0,36-1,37	0,307^1^
Por profissional de saúde	31 (42,5)	42 (57,5)	1,00		
**Recebeu orientação profissional para o uso?**					
Sim	30 (43,5)	39 (56,5)	1,50	0,77-2,95	0,228^1^
Não	26 (33,8)	51 (66,2)	1,00		

1 Teste qui-quadrado de Pearson;

2 Razão de verossimilhança; RC - Razão de Chance; IC95% - Intervalo de Confiança de 95%.

Pode-se verificar maior frequência de alto letramento em saúde entre as solteiras com parceiro fixo (84,2%; *p*=0,116; RC=2,0; IC=0,9-4,8), entre as jovens que tiveram gestações na adolescência em suas famílias (60,2%; *p*=0,689; RC=1,14; IC=0,5-2,2), assim como entre as participantes que não engravidaram (62,2%; *p*=0,317; RC=3,2; IC=0,2-37,2). Com relação ao tipo de anticoncepcional hormonal utilizado, os três métodos apresentaram maioria das participantes com alto letramento.

A partir dos valores de *p* do teste de correlação de Spearman, quando cruzados com as variáveis numéricas (Tabela 4), verificou-se que a pontuação total da HLS-14 se correlacionou, de maneira inversamente proporcional, somente à variável idade (rs=-0,218; *p*=0,008).

**Tabela 4:** Análise de correlação linear pelo coeficiente de correlação de Spearman entre o valor da *Health Literacy Scale*-14 e as variáveis numéricas, Fortaleza, Ceará, Brasil, 2020

Variáveis	Pontuação total da HLS-14	
	rs	Valor de *p*
Qual sua idade? (n=146)	-0,218	**0,008**
Quantas pessoas moram na residência? (n=146)	-0,045	0,591
Com quantos anos foi sua primeira menstruação? (n=144)	0,021	0,798
Tempo de uso do método (atual) em meses (n=135)	-0,160	0,063

*rs - rô de Spearman; HLS-14 - Health Literacy Scale-14.*

## DISCUSSÃO

A amostra da pesquisa foi composta por adolescentes sexualmente ativas e que faziam uso de anticoncepcional hormonal, observando-se o início precoce das relações sexuais nas meninas a partir dos 15 anos de idade. Obteve-se média de idade maior do que a encontrada no PeNSE-2019, que avaliou estudantes de escolas públicas e particulares de todo o território nacional, e apontou que 35,4% dos estudantes entre 13 e 17 anos tiveram relação sexual alguma vez, com média de idade da primeira relação sexual de 13,8 anos^(3)^.

O não uso de métodos contraceptivos, o fato de estar solteira e a falta de apoio do pai da criança estiveram associados à ocorrência de gravidez na adolescência em pesquisa realizada com 518 mulheres grávidas de São Tomé e Príncipe, um país da África Central, em que, destas, 20,1% eram adolescentes^(18)^. No presente estudo, 50,3% das participantes afirmaram utilizar o anticoncepcional oral, e 25,9% relataram fazer uso da pílula de emergência como método contraceptivo, apresentando uma porcentagem preocupante de adolescentes que utilizam como método regular a pílula do dia seguinte, o que compromete sua eficácia.

O uso inconsistente/inadequado do método contraceptivo eleva sua taxa de falha e a chance de uma gravidez indesejada, aumentando os riscos de morbimortalidade materna, conforme pesquisa realizada na Nigéria^(19)^. Em estudo realizado no Quênia, encontrou-se que as adolescentes e mulheres jovens (15 a 24 anos) tinham risco 1,2 vezes maior de interromper o uso de um método contraceptivo, e apresentaram um aumento de 20% no risco de descontinuação de um método no decorrer do período analisado, quando comparado com o grupo de mulheres com mais de 35 anos^(20)^.

Diante do exposto, perguntou-se de que maneira as adolescentes têm compreendido as informações que recebem, quais fontes de informação elas têm buscado e quais embasamentos elas têm se apropriado para tomar decisões. Para prevenir uma gestação indesejada e/ou não planejada na adolescência, são necessários acesso à informação correta, orientação adequada e julgamento crítico para direcionar a tomada de decisão.

No presente estudo, a maioria das adolescentes (80,3%) apresentou letramento em saúde alto, fato que favorece a adoção de medidas preventivas e comportamentos de saúde seguros. De modo semelhante, em pesquisas realizadas na Noruega e na Coreia do Sul, os adolescentes apresentaram níveis altos e moderados de letramento em saúde^(21,22)^. Nesses estudos, o letramento em saúde se associou às medidas de proteção à saúde e aos comportamentos saudáveis relacionados à pandemia de COVID-19.

Por outro lado, estudo realizado na Etiópia com 577 estudantes do ensino médio encontrou que a maioria dos adolescentes possuía conhecimento precário sobre o uso de preservativos e doenças sexualmente transmissíveis, e 69,6% dos adolescentes possuíam escore problemático ou inadequado de letramento em saúde sexual e reprodutivo, similar ao observado em pesquisa brasileira, em que 69,44% dos escolares apresentaram níveis baixos de letramento em saúde relacionado aos métodos contraceptivos^(23,24)^.

As adolescentes deste estudo apresentaram baixo nível de letramento funcional, porém alto nível de letramento comunicativo e crítico. Essa situação se caracteriza por baixa habilidade na leitura, na compreensão das informações escritas e na compreensão das orientações transmitidas oralmente por profissionais da saúde, fato que prejudica a tomada de decisão, reverberando no uso incorreto de medicações, por exemplo^(17)^.

As habilidades da leitura e numeracia, componentes do letramento funcional, são essenciais na aquisição do letramento em saúde, envolvendo as habilidades básicas de captação das informações. Na Indonésia, em estudo com 1.066 estudantes entre 14 e 19 anos, apenas 25% dos participantes possuíam alto nível de letramento funcional em saúde, e os escores adequados dessa dimensão se associaram aos comportamentos de saúde de não uso de álcool, drogas e cigarro^(25)^.

Pesquisa realizada com 288 adolescentes brasileiros, em um município do estado do Pará, observou que a maioria alegou dificuldade para compreender as orientações fornecidas de forma escrita, além de problemas para entender o conteúdo de materiais educativos sobre métodos contraceptivos. Além disso, 58,6% também referiram dificuldade para compreender as orientações verbais fornecidas por profissionais de saúde^(24)^.

Com relação à idade, verificou-se correlação inversamente proporcional com o letramento em saúde (rs=-0,218; *p*=0,008), em que as adolescentes entre 18 e 19 anos tinham 2,69 mais chances de apresentarem baixo letramento, quando comparadas com as mais jovens (*p*=0,005). Esse achado é consistente com o de estudos anteriores realizados em estados da região Sudeste do Brasil, em que a menor idade esteve associada a maiores níveis de letramento funcional em saúde, juntamente com maior escolaridade e alta renda^(26,27)^.

De forma semelhante, estudo realizado na China com grupos de diferentes idades encontrou que o letramento em saúde declinou conforme o avanço da idade, com os adolescentes apresentando melhores níveis de letramento quando comparados com as outras faixas etárias^(28)^. Com um contato mais frequente com a internet e redes sociais, os jovens, em geral, apresentam maiores chances de buscar e encontrar informações de saúde, porém maior dificuldade em avaliar sua veracidade e segurança, o que influencia o nível de letramento em saúde^(24,29)^.

Quanto às variáveis familiares, as adolescentes apresentaram maior chance de baixo letramento quando o chefe da família (a pessoa que tinha o maior salário) não era o pai ou a mãe (RC=2,83; *p=*0,012), e quando não residiam com nenhum dos genitores (RC=2,77; *p*=0,009).

Em pesquisa realizada com adolescentes e jovens do sexo feminino, no Mato Grosso, Brasil, as participantes que não pertenciam às famílias nucleares tinham mais chance de engravidar na adolescência quando comparadas às jovens provenientes de famílias com ambos os pais^(30)^. Essa composição familiar também esteve associada ao comportamento sexual de risco, à iniciação sexual precoce em estudo nacional realizado com adolescentes brasileiros^(31)^ e ao consumo excessivo de álcool em jovens de município baiano^(32)^.

Com relação ao uso do anticoncepcional, a única variável que apresentou associação significante com o letramento em saúde foi o tempo de uso do método, em que as adolescentes que utilizavam contraceptivo há mais tempo tinham 2,68 vezes mais chances de terem baixo letramento do que as que utilizavam há menos tempo (*p*=0,007). Fato similar é encontrado em estudo realizado com 52 mulheres usuárias de anticoncepcionais hormonais injetáveis, em que a maioria das mulheres em uso inseguro do método estava utilizando há mais de 12 meses^(33)^.

O tempo de uso de métodos contraceptivos e a descontinuação estiveram relacionados a vários fatores, como falta de acesso, pouco conhecimento sobre como lidar com o método e surgimento dos efeitos colaterais^(20,33)^, sendo fundamentais a condução e a orientação adequadas durante as consultas de planejamento reprodutivo quanto ao manejo do método que garantam a compreensão do uso correto por parte da paciente.

Estudos mostram que pessoas que possuem maior letramento em saúde estão mais propícias a aderir comportamentos mais saudáveis, por possuírem capacidade maior de compreensão das orientações que lhes foram passadas, como prescrição, marcação de consulta, rótulos de medicações e outras informações para o autocuidado^(16)^.

### Limitações do estudo

As limitações do estudo estão relacionadas ao tamanho da amostra e à menor abrangência de idades, que se deram em virtude das dificuldades na coleta advindas da situação de pandemia, o que dificulta a generalização do estudo para escolas com diferentes realidades. Além do mais, observou-se resistência por parte de alguns diretores das escolas na participação da pesquisa, em decorrência do tema relacionado ao uso dos métodos contraceptivos, pois se relatou receio dos pais considerarem como incentivo à prática sexual, fato que também contribuiu para diminuir a adesão à pesquisa por parte das adolescentes.

### Contribuições para as áreas da enfermagem, saúde ou políticas públicas

Compreender os fatores que contribuem para o uso correto dos anticoncepcionais permite aos profissionais de saúde a elaboração de estratégias de comunicação que facilitem a tomada de decisão informada e estimulem uma maior autonomia no processo de cuidado pelo público jovem. Estudos desta natureza ampliam o repertório de conhecimentos para o fortalecimento da enfermagem na prática baseada em evidências, com destaque para a utilização de estratégias educativas que promovam a utilização e adesão adequadas dos métodos contraceptivos por parte das adolescentes.

## CONCLUSÕES

As adolescentes participantes apresentaram alto nível de letramento em saúde, o que reflete sua capacidade de buscar e avaliar as informações sobre saúde reprodutiva. Entretanto, em contraste, foi observado que elas iniciaram o uso de anticoncepcional hormonal e a vida sexual com déficit no letramento funcional, ou seja, baixa habilidade de leitura e compreensão das informações recebidas sobre o método. Essa lacuna representa um fator de risco significativo, pois pode levar à interpretação equivocada de orientações, ao uso incorreto do anticoncepcional e, consequentemente, ao aumento da probabilidade de gravidez indesejada e/ou não planejada.

Embora tenham demonstrado bom desempenho nos níveis comunicativo e crítico, a deficiência no letramento funcional limita sua autonomia e autocuidado. O baixo letramento em saúde se associou às variáveis idade, composição familiar (chefe da família, com quem mora) e tempo de uso do método contraceptivo, sugerindo que, além de fatores intrínsecos, ambiente familiar e experiência prévia também afetam os comportamentos de saúde.

Diante desses achados, torna-se urgente avançar nas pesquisas sobre letramento em saúde de adolescentes no Brasil, não apenas no contexto do uso de anticoncepcionais, mas também em outras temáticas relevantes para esse público, objetivando orientar e embasar as práticas profissionais na enfermagem e na saúde pública, e permitindo a criação de estratégias educativas adaptadas a eles.

## Data Availability

Os dados de pesquisa estão disponíveis em repositório: https://osf.io/jmhbg/?view_only=ab0331e8f13e4ff0b0cb232fb68c6c21.
